# The Osteoarthritis Natural Progress and Changes in Intraosseous Pressure of the Guinea Pig Model in Different Degeneration Stages

**DOI:** 10.1111/os.13496

**Published:** 2022-09-28

**Authors:** Shuo Wang, Jianxiong Ma, Xingwen Zhao, Yuan Xue, Wei Liu, Hongchao Huang, Lei Zhang, Aixian Tian, Xinlong Ma

**Affiliations:** ^1^ Orthopaedics Institute of Tianjin, Tianjin Hospital Tianjin University Tianjin China; ^2^ Department of Orthopaedics Chinese People's Liberation Army Joint Logistics Support Force Tianjin Rehabilitation Center (Former No. 464 Hospital of People's Liberation Army) Tianjin China; ^3^ Department of Orthopaedics Tianjin Medical University General Hospital Tianjin China; ^4^ Department of Orthopaedics Tianjin Baodi Hospital Tianjin China; ^5^ Department of Orthopaedics Tianjin Medical University Hospital for Metabolic Diseases Tianjin China

**Keywords:** Articular cartilage, Degeneration, Intraosseous pressure, Knee osteoarthritis, Subchondral bone

## Abstract

**Objective:**

Articular cartilage and subchondral bone changes during the pathological progress of knee osteoarthritis (KOA) is a key event marking the development of the disease. The age varying alteration patterns within entire osteochondral unit remains poorly understood. The purpose of this study was to find a reasonable age range of the Dunkin–Hartley guinea pig model for the studying of KOA pathological process, and to investigate Intraosseous pressure (IOP) in the process during different degeneration stages of KOA.

**Methods:**

Male Dunkin–Hartley guinea pigs were selected and divided into groups of 3, 6, 9, 12, 18 months old by age, 10 in each group. All knees underwent imaging examination including X‐ray, Micro‐CT and MRI. Observed the imaging findings with the use of Kellgren–Lawrence (K‐L) classification and knee osteoarthritis MRI scores. Measured the IOP of distal femur (DF) and proximal tibia (PT) in each group, and observed the differences of bilateral tibiofemoral articular cartilage in histological and immunohistochemistry, staining results were evaluated by using Mankin's score. Analysis of variance (ANOVA) and *t*‐tests were used to compare the differences indicators between groups.

**Results:**

With the increase of age, changes in X‐ray, Micro‐CT and MRI imaging findings and pathological staining results of articular cartilage in all stages were consistent with the changing of degenerative KOA process. The IOP of DF and PT increased gradually with age, and reached its peak in 12‐month age group, and then gradually decreased, there was a statistically significant difference of IOP between each group. The IOP of DF was slightly higher than that of PT, but the difference was not statistically significant.

**Conclusion:**

Dunkin–Hartley guinea pigs can be used as an animal model to study different pathological stages of KOA. There might be a correlation between the changes of IOP and the pathological progress of articular cartilage and subchondral bone in DF and PT.

## Graphical abstract

Posture for intraosseous pressure measurement (IOP). Puncture needle was punctured from the medial side direct to the opposite side of the lateral cortex and connected to the pressure measuring tube. After IOP measuring.

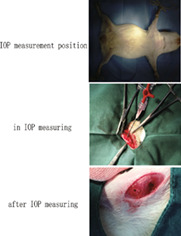

## Introduction

Knee Osteoarthritis (KOA) is a late‐onset, non‐specific joint‐damage disease characterized by articular cartilage degeneration, subchondral bone sclerosis, bone cyst, and cartilage marginal callus formation.^1^, ^2^ Epidemiological investigation confirmed that the incidence of KOA is related to age, trauma, obesity, and genetic factors,^2^, ^3^, ^4^ but the etiology remains unclear. The biopsy of weight‐bearing articular cartilage in time series is contrary to the ethical guidelines for human trials. Therefore, it is very necessary to find suitable experimental animal models for etiological study.

At present, the establishment of the KOA model is often induced by surgical means, such as knee meniscus resection or anterior cruciate ligament disconnection.^5^, ^6^, ^7^ But osteoarthritis induced by traumatic procedure does not conform to the mechanism of natural degeneration. The sequence of degeneration and instability is totally opposite during the development of natural degenerative and traumatic arthritis. The cause of KOA which is induced by human intervention is clear and not suitable for degenerative KOA research.^6^, ^7^

However, the KOA of Dunkin–Hartley guinea pigs naturally occurs. The degree of lesions is positively related to age and weight. This animal model does not require special modeling, it has the characteristics of docile, economical, easy to raise and management.^7^ Imaging findings shows symmetric articular cartilage lesions outside the meniscus and severe involvement of the medial tibia plateau which are very similar to those of human degenerative KOA lesions. Therefore, Dunkin–Hartley guinea pigs are suitable as an experimental animal model for degenerative KOA research.^7^, ^8^ But the reasonable age range for the complete coverage of degenerative KOA pathological process is not clear.

Intraosseous pressure (IOP) refers to the pressure generated within the marrow space. This concept is used to describe the disorder of the circulatory state in bone.^9^ Intraosseous decompression has been clinically proven to effectively relieve the rest pain in KOA patients and to some extent improve knee function. There is obvious bone marrow edema in the middle and late stage of KOA, and this may be associated with local inflammatory reaction and intramedullary venous return disorder,^10^ thereby inducing an increase of IOP. Therefore, IOP might have a positive correlation with KOA pathological process.

Therefore, Dunkin–Hartley guinea pigs of different ages were involved in this study. On the one hand, the standardized age range was confirmed by imaging and histology. On the other hand, the pathological process of degenerative KOA was observed by introducing the concept of IOP. The purpose is: (i) To determine the age range corresponding to the different pathological stages of degenerative KOA, in order to accurately select the suitable age of guinea pigs during KOA research; and (ii) To observe the changing characteristics of IOP in the progress of degenerative KOA.

## Methods

Male Dunkin–Hartley guinea pigs of 3, 6, 9, 12, and 18 months old were randomly selected, 10 for each month age. All Dunkin–Hartley guinea pigs were housed in a specific pathogen‐free animal room at a temperature of 18–22 °C, humidity 40%–70%, and 12‐h circadian rhythm.

The animal experiments involved in the study were approved in advance by the Experimental Animal Care and Use Committee (EACUC) of Tianjin Hospital (2021–057). We measured the body weight (g) of DH guinea pigs in each month‐age group, and drew the weight‐month‐age curve of different month‐age groups.

### Tibiofemoral Joint (TFJ) Imaging Examination

#### X‐Ray Evaluation

Ten per cent chloral hydrate solution was intraperitoneally anesthetized, and the positive lateral radiographs of both knees were taken by UltraFocus DXA (Faxitron, Tucson, AZ, USA). The degree of knee degeneration was determined by Kellgren–Lawrence (K‐L) classification (Table S1).^11^

#### MRI Evaluation

In a Supine position, the wrist joint coil was used to perform double‐knee MRI examination (Discovery™ MRI750 3.0T system, General Electric Company, Lincoln, NE, USA). Since there is no MRI scoring system for small animal knee osteoarthritis, a MRI simple scoring system for guinea pig knee osteoarthritis was developed with reference to the rheumatoid MRI scoring system (RAMRIS).^12^ The system consists of three parts, including synovitis (0–2 points), bone marrow edema and/or cystic changes (0–2 points), with a minimum score of 0 and a maximum score of 6 (Table S2). MRI results were determined by two experienced radiologists in a single blind method. The two radiologists only knew that it was the knee joint of the Dunkin–Hartley guinea pig, but did not know the specific age of the guinea pig.

#### Micro‐CT Scan

All guinea pigs were euthanized and bilateral knee joints were taken for micro‐CT scanning (Inveon preclinical small‐animal PET/SPECT/CT system, Siemens, Frankfurt, Germany) with 8.5 μm scanning accuracy, 80 kV and 500μA.

The anatomical region of interest (ROI) was the weight‐bearing area of the patellofemoral joint. The coronal plane was marked from the anterior calcified cartilage of meniscus to the sesamoid of posterior‐superior femoral condyle. The sagittal plane was marked between medial and lateral crest of femoral patella surface (Figure S1).

#### Measurement of IOP

**Fig. 1:**
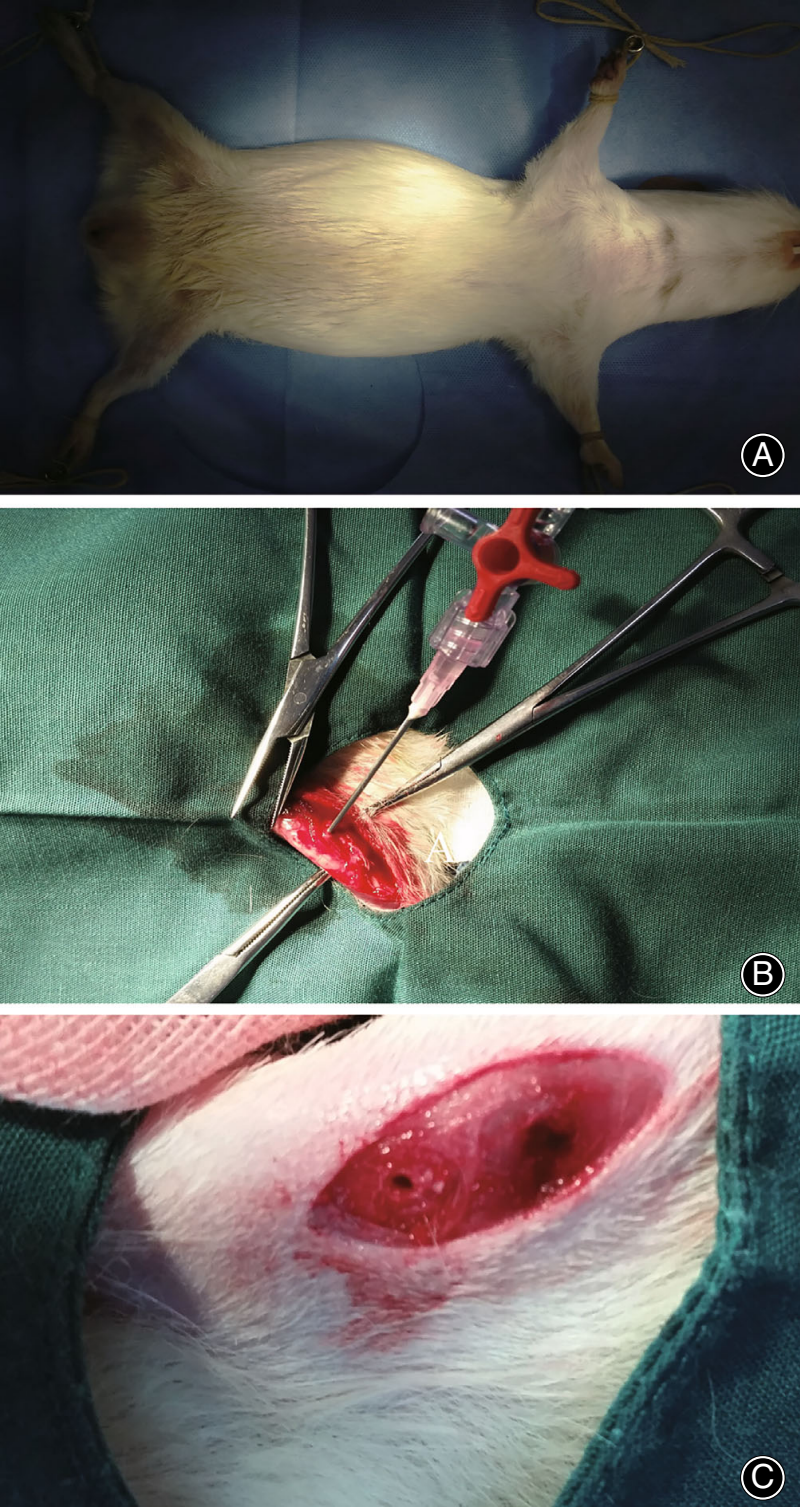
(A) Posture for intraosseous pressure measurement (IOP). (B) Puncture needle was punctured from the medial side direct to the opposite side of the lateral cortex and connected to the pressure measuring tube. (C) After IOP measuring

Posture for intraosseous pressure measurement was supine, with both lower limbs abducted, flexed hips and knees (Fig. 1). Checked the tightness of the pressure measuring pipeline, connected the pressure measuring tube and zeroed the dashboard. A puncture needle was used to puncture into the distal femur (DF) and proximal tibia (PT) bone marrow cavity and blood return was noted. The medial collateral ligament attachment point at both DF and PT were selected as the puncture point, because the position of the attachment points was relatively fixed, ensuring the accuracy and repeatability of the pressure measurement. A Puncture needle (Ø 0.45mm, Ø 0.7mm, Ø 0.8mm, Hanaco Medical Co. Ltd., Tianjin, China) was used from the medial side direct to the opposite side of the lateral cortex (depth of penetration), the diameter of the puncture hole was 0.7 mm. Then the puncture needle was connected to the pressure measuring tube (Ø 1.8 mm, Plastics Research Institute, Tianjin, China). Through the CSY‐B ceramic thick film pressure sensor, the final pressure was visible on the display (Saiying Electronics Technology Development Inc., Bengbu, China).

#### Histological Evaluation

The general specimen was observed and the glossiness, integrity and osteophyte hyperplasia of the articular cartilage was evaluated. Two experienced pathologists simultaneously evaluated the general specimen. Articular cartilage of bilateral tibiofemoral joints (TFJ) were stained with HE, toluidine blue, Type 2 collagen and MMP13 immunohistochemical staining. The degeneration degree of the articular cartilage matrix was evaluated by type 2 collagen staining, and the inflammatory response in the cartilage matrix was evaluated by the expression of MMP13. The immunohistochemical staining was performed by Strept Avidin‐Biotin Complex (SABC) method. Knee cartilage pathological staining scores were performed by using semi‐quantitative Mankin's score.^13^ But for Col‐2a and MMP13, only the matrix staining section was used in the Mankin's score for scoring and statistics (Table S3).^14^

### Statistical Analysis

A one‐way ANOVA was used for analyzing the difference of Dunkin–Hartley body weight‐month‐age changes, knee arthritis MRI score, Mankin's score, DF and PT IOP changes in each group. The *t*‐test was used for comparison between groups. The test level was taken as both sides α = 0.05.

## Results

### Weight‐Month‐Age Curve

**Fig. 2:**
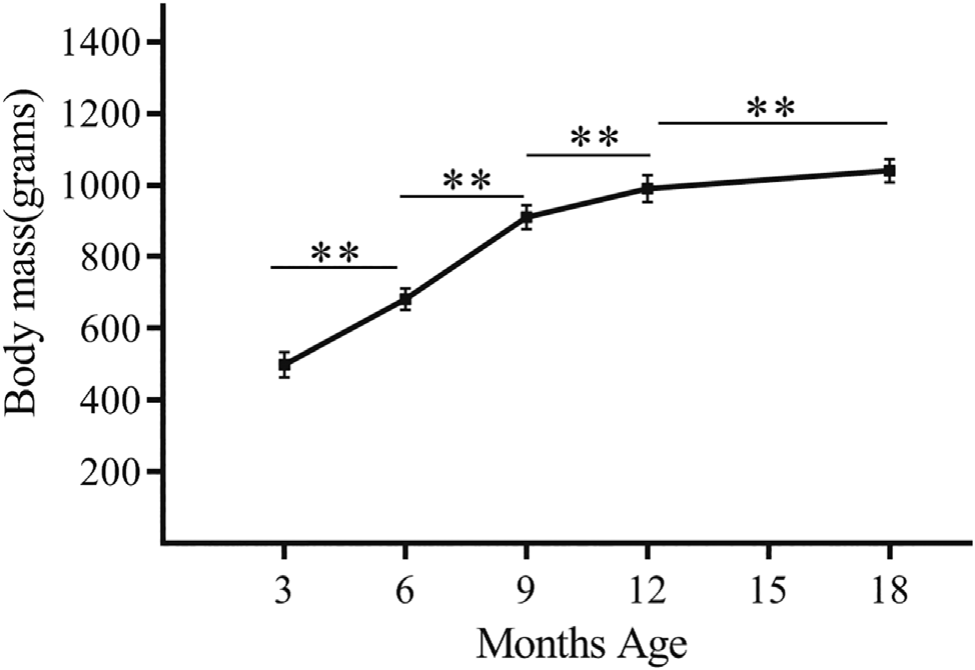
Weight‐month‐age curve

The weight of Dunkin–Hartley guinea pigs gradually increased with the age of months. Before the age of 9 months, the weight gain was relatively rapid, but after 9 months, the weight gain gradually slowed down. The difference in weight between the groups was statistically significant (Fig. 2, F = 433.892，*P* = 0.000).

### X‐Ray Performance of Bilateral TFJ

**Fig. 3:**
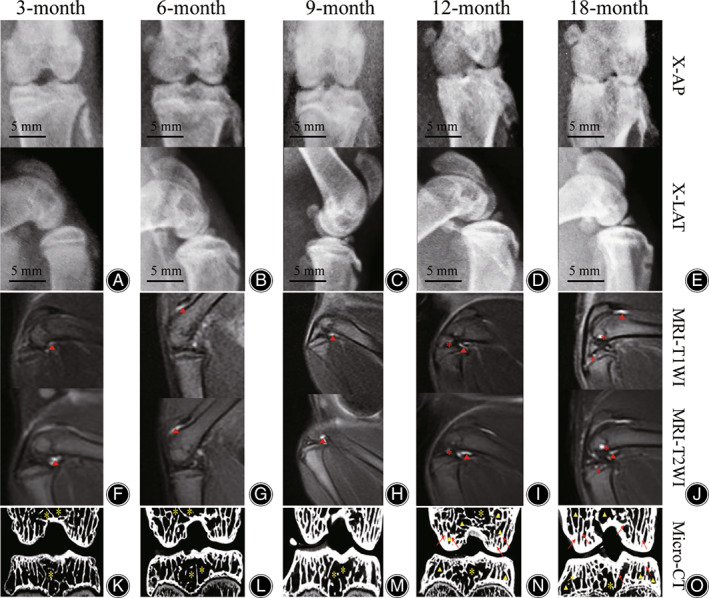
(A–E) X‐ray images of right knee joints in each age group (AP, anteroposterior view; LAT, lateral view). (F–J) MRI images of right knee joints in each age group (Triangle indicated synovitis‐like changes; asterisk indicated BMLs of subchondral bone). (K–O) Micro‐CT images of right knee joints in each age group (Triangle indicated subchondral bone cysts; asterisk indicated density reduction zone; arrow indicated hardened cancellous bone)

In the 3‐month age group, there was no osteophyte formation of bilateral TFJ and K‐L grade were all 0. in the 6‐month age group, small osteophyte was not seen or faintly visible at medial tibial plateau, and K‐L grade was 0 in seven cases, 1 in three cases. In the 9‐month age group, small osteophyte was faintly visible at tibial plateau and intercondylar ridge, and K‐L grade was 1 in three cases, 2 in seven cases. In the 12‐month age group, the tibial plateau showed obvious osteophytes, partial TFJ space stenosis, subchondral bone sclerosis and cystic changes, and K‐L grade was 2 in two cases, 3 in eight cases. In the 18‐month age group, the tibial plateau showed obvious osteophytes, subchondral bone sclerosis and cystic changes, and the bone ends were deformed, and K‐L grade were all 4. It can be seen that with the increase of age, the K‐L grade gradually increased, suggesting that osteoarthritis gradually worsened (Fig. 3A–E).

### MRI Findings of the Knee Joint

In the 3‐month and 6‐month age groups, the medial posterior intercondylar fossa of femur and patellofemoral joint space were both long T1 and long T2 signals, showing synovitis‐like changes, but there was no significant difference between the two groups. In the 9‐month age group, synovitis‐like changes were located in the medial posterior side of intercondylar fossa of femur, and the signal was stronger than the 3‐month and 6‐month age groups (Figure 3F–J). In the 12‐month age group, on the basis of synovitis‐like changes, there was a further high‐signal area of the subchondral bone in the DF and PT, which suggesting bone marrow lesions (BMLs). In the 18‐month age group, synovial‐like signal intensity was significantly weaker than that of 12‐month age group, while BMLs in the DF and PT were significantly enhanced compared with the 12‐month age group.

**Fig. 4:**
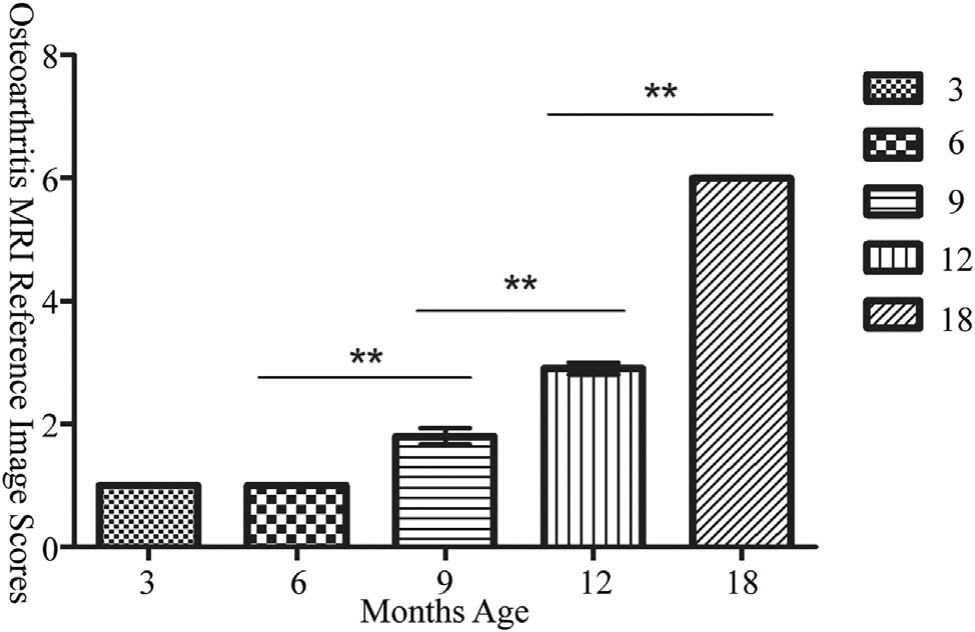
MR scores

The MRI scores of KOA were consistent with the 3‐month and 6‐month age groups, and the difference was not statistically significant (*P* = 1.000). Moreover, the score increased gradually after 6 months. The average score was 1.0 ± 0.000 point at 3 and 6 months, 1.8 ± 0.422 points at 9 months, 2.9 ± 0.316 points at 12 months, 6.0 ± 0.000 points at 18‐months. The difference was statistically significant between each group (Figure 4, *F* = 782.640, *P* = 0.000).

### Micro‐CT Performance of TFJ

In the 3‐month and 6‐month age groups, microscopic callus formation was observed in the femoral condyle and PT. No typical subchondral bone cyst formation was observed, but the density reduction zone was seen between the DF and the PT. In the 9, 12 and 18 months age groups, the thickness of the subchondral bone plate and the width of the trabecular bone increased significantly. With the increase of age, the density reduction zone between the DF and the PT was gradually surrounded by trabecular bone. In the 18‐month age group, typical subchondral bone cyst formation can be seen, which was consistent with MRI intramedullary signal characteristics (Fig. 3K–O).

Subchondral bone cysts can be observed at both 12‐month and 18‐month age group, which were located at the DF and PT. They showed a hardened cancellous bone which was adjacent around the subchondral bone plate and was thickened and not adapted to mechanical conduction. And the typical interior has no trabecular bone structure (Fig. 3K–O).

With the increase of age, the subchondral trabecular bone in the weight‐bearing area can collapse and fuse into pieces or clumps, and can merge with the adjacent subchondral bone plate. This phenomenon was found in the DF and the PT weight‐bearing area at 9, 12 and 18 months(Fig. 3K–O). Subchondral bone cyst and subchondral trabecular collapse fusion are very similar to human KOA subchondral bone changes.

### Histology Results of TFJ

#### General Result

**Fig. 5:**
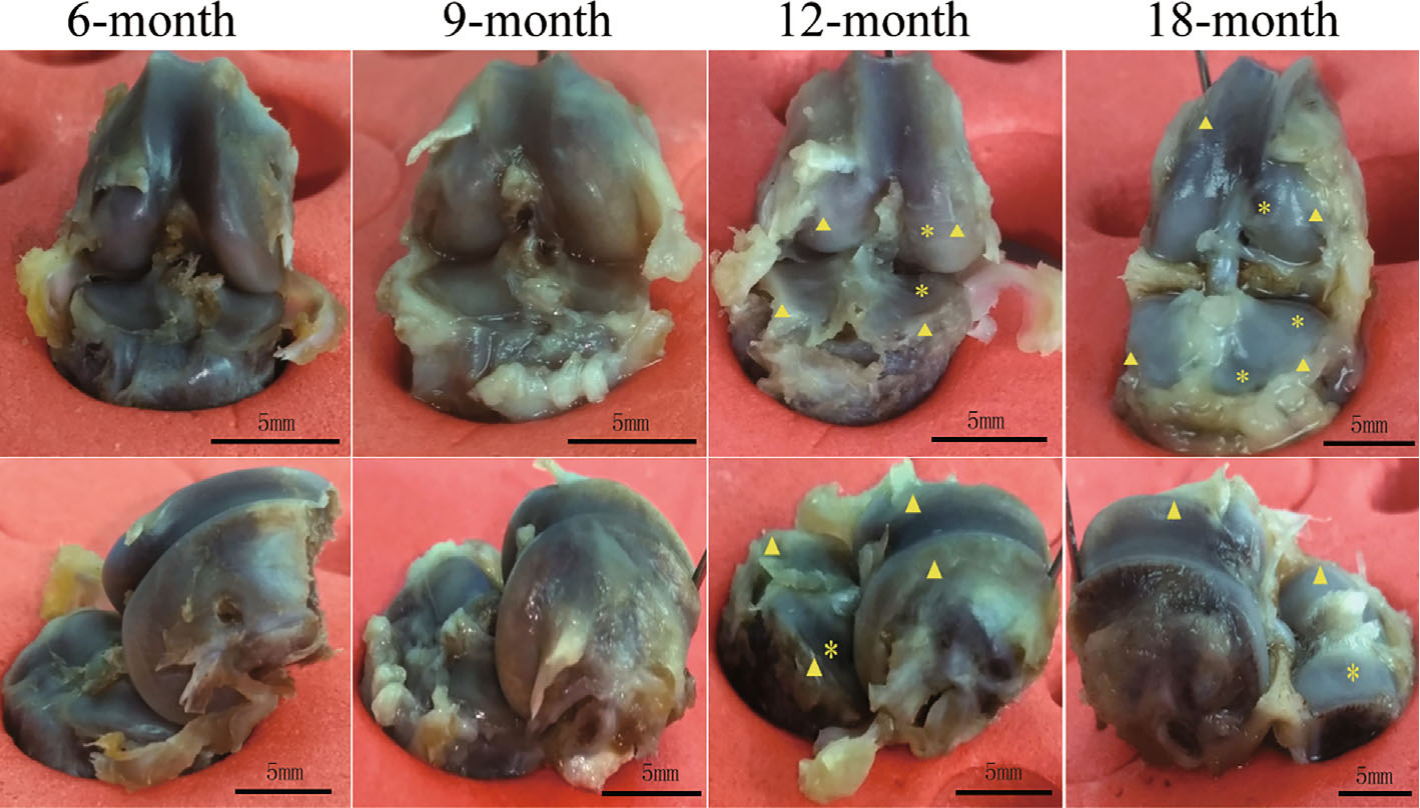
General observation results of each age group specimen (Triangle indicated wax‐like osteochondral hyperplasia; asterisk indicated cartilage defects; DF, distal femur; PT, proximal tibia)

In the 6‐month age group, articular cartilage had good gloss and transparency, no obvious cartilage defects were found in the distal femoral and PT articular surface weight‐bearing areas, the trochlear crest of femur and PT appeared to have cartilage‐like hyperplasia; In the 9‐month age group, the articular cartilage had good gloss and transparency, but lower than that of the 6‐month age group, the trochlear crest of femur was blunt, obvious cartilage‐like hyperplasia can be seen at DF and PT; In the 12‐month age group, articular cartilage was turbid, visible cartilage surface defects were found in the DF and the PT articular surface weight‐bearing area, and the defects of medial condyle were particularly obvious. Visible wax‐like cartilage or bone‐like hyperplasia were observed at the DF and PT. The “U”‐shaped cartilage defect in the posterior part of medial condyle of PT was formed, corresponding to the turbid color of the cartilage in the medial condyle of femur; In 18‐month age group, articular cartilage was turbid, except for the DF and PT cartilage surface defect, partial subchondral bone was exposed at the edge of the tibial plateau. The DF and the proximal edge of the tibia showed obvious wax‐like osteochondral hyperplasia. The “U”‐shaped cartilage defect in the posterior part of medial condyle was extended to the middle and front, and the cartilage was significantly thinner (Fig. 5).

### Eosin–Hematoxylin Staining

**Fig. 6:**
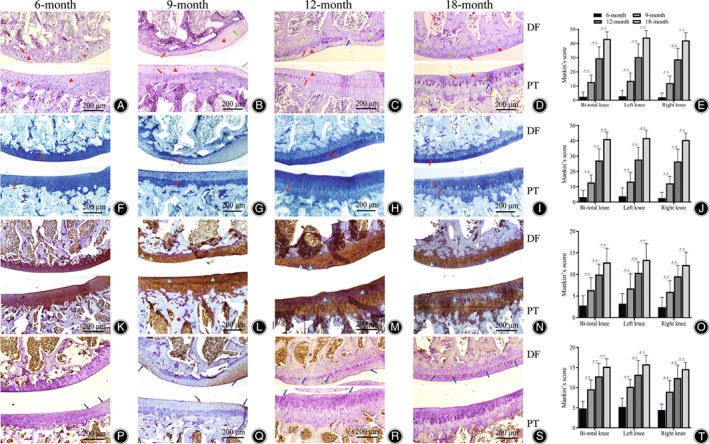
(A–D) HE staining of right knee joint in each age group (Triangle indicated cartilage matrix; red arrow indicated tidal line; green arrow indicated vacuolar‐like degeneration; blue arrow indicated cartilage fissure, yellow arrow indicated blood vessel; × 40). (E) Mankin's score of HE staining. (F–I) Toluidine blue staining of right knee joint in each age group (red arrow indicated tidal line; × 40). (J) Mankin's score of toluidine blue staining. (K–N) Col‐2a staining of right knee joint in each age group (triangle indicated staining reduction zone; × 40). (O) Mankin's score of Col‐2a staining. (P–S) MMP13 staining of right knee joint in each age group (arrow indicated MMP13 positive expression region; × 40). (T) Mankin's score of MMP13 staining. (DF, distal femur; PT, proximal tibia; values shown are means ± SD. Double asterisk (**) indicate significant differences (*P* < 0.05) at right knee or left knee, respectively)

In the 6‐month age group, articular cartilage showed normal or mild degeneration. Normal or reduced cartilage matrix staining was limited to the cartilage surface; the distribution of chondrocytes was normal or slightly increased; the tidal line was intact (Fig. 6A–D).

In the 9‐month age group, articular cartilage showed mild degeneration. The cartilage surface was slightly irregular; normal or reduced cartilage matrix staining was limited to the cartilage surface; chondrocytes can be locally enlarged, vacuolar‐like degeneration, or even partial full‐thickness reduction; the tidal line was intact (Fig. 6A–D).

In the 12‐month age group, articular cartilage can be degenerated at various stages, with moderate degeneration being the most common. The cartilage fissure or loss extended to the middle layer or even the deep layer. The reducing of cartilage matrix staining was limited to the surface layer or extended to the middle layer, and the medial condyle was most obvious; chondrocytes can be locally increased or decreased, while chondrocytes of medial condyle were reduced; intact tidal line, intersecting with blood vessels or multiple tidal lines can be observed (Fig. 6A–D).

In the 18‐month age group, articular cartilage can be degenerated at moderate–severe stages, with severe degeneration being the most common. The cartilage fissure or loss located in the middle layer or extended to the deep layer. The reducing of cartilage matrix staining extended to the middle layer or even the deep layer, and the medial condyle was most obvious; chondrocytes showed diffuse increased or focal decreased; incomplete tidal line, intersecting with blood vessels or multiple tidal lines can be observed (Fig. 6A–D).

The Mankin's score increased with age, and the difference between the groups was statistically significant (*F* = 101.490, *P* = 0.000). Compared with the bilateral scores in each age group, the difference was not statistically significant. (*P* > 0.05, Fig. 6E).

### Toluidine Blue Staining

The staining results of each age group were consistent with HE staining (Fig. 6F–I). The Mankin's score increased with age, and the difference between the groups was statistically significant (*F* = 90.405, *P* = 0.000). Compared with the bilateral scores in each age group, the difference was not statistically significant. (*P* > 0.05, Fig. 6J).

### Type 2 Collagen Immunohistochemical Staining

In the 6‐month age group, the distribution of Col‐2a in articular cartilage was clear, and the focal staining of the lateral condyle cartilage weight‐bearing area was reduced in the middle layer, and extended to the deep layer in medial condyle. The degree of coloration was coupled with cell proliferation, and the higher the degree of local cell proliferation, the weaker the positive staining of Col‐2a (Fig. 6K–N).

In the 9‐month age group, the reduction of Col‐2a staining in articular cartilage was characterized by irregular distribution. The lateral condyle reduction zone was located in the surface layer and middle layer of cartilage, and reached to the deep layer in medial condyle, often accompanied by a decrease in the number of local cells. It was worth noting that the reduction of Col‐2a staining may result in a jump change, that was, the deep and middle layer staining were reduced, while the surface layer was still continuous positive (Fig. 6K–N).

In the 12‐month age group, the degree of reduction and involvement of the articular cartilage Col‐2 staining was significantly aggravated compared with the 6 and 9 months' age groups. The staining reduction zone was strip or sheet shape. The lateral condyle mainly involved the surface layer, middle layer and even deep layer of cartilage, and the medial condyle obviously involved the deep layer and even the whole layer. There were also cases where the middle layer and deep layer staining reduced, while the surface layer staining enhanced (Fig. 6K–N).

The 18‐month age group, the degree of reduction and involvement of the articular cartilage Col‐2 staining was further aggravated compared with the 12‐month age group.

The staining reduction zone could involve the whole layer of articular cartilage, mainly the middle and deep layers. There was no significant difference in the depth of cartilage involved of internal and external condyle (Fig. 6K–N).

With the increasing of age, the Mankin's score gradually increased, and the difference between each age group was statistically significant (*F* = 25.399, *P* = 0.000), and the difference between the groups of 6, 9 and 12 months' age was significant (LSD‐t: 6 *vs* 9, *P* = 0.006; 9 *vs* 12, *P* = 0.006), there were statistical differences between the 12 and 18 months' age group (*P* = 0.028, Fig. 6O); there was no significant difference in the bilateral scores of the same age group (*P* > 0.05).

### MMP13 Immunohistochemical Staining

In the 6‐month age group, the positive MMP13 mainly accumulated in DF and PT, the lateral edge of cartilage and the surface layer of articular cartilage. In the middle and deep layers, MMP13 was scattered in the cells, and distributed in cells and matrix in the surface layer, which was consistent with synovitis (Fig. 6P–S).

in the 9‐month age group, positive MMP13 was disseminated by the intercondylar and cartilage lateral margins to the central weight‐bearing area, showing a map‐like distribution, mainly involving the surface layer and intermediate layer of the articular cartilage, and can also affect the whole layer. When the middle layer and the deep layer were involved, there were positive expressions in both cells and matrix (Fig. 6P–S).

In the 12‐month age group, positive MMP13 showed scattered distribution of articular cartilage, but the expression decreased, which may be related to cell degeneration, necrosis, shedding and cartilage matrix degradation. Positive MMP13 was found in cartilage exfoliation, indicating that cartilage destruction was in progress (Fig. 6P–S).

In the 18‐month age group, positive MMP13 showed a full‐layer distribution of articular cartilage, and MMP13 line‐like deposition occurred between the middle layer, deep layer and calcified layer. This phenomenon may reflect the pathological mechanism of cartilage exfoliation (Fig. 6P–S).

With the increase of age, the Mankin's score increased gradually. There was significant statistical difference between each age group (I = 35.120, *P* = 0.000), and the difference between groups was significant between 6, 9 and 12 months (LSD‐t: 6 *vs* 9, *P* = 0.000; 9 *vs* 12, *P* = 0.005), the difference between 12‐month and 18‐month age group was statistically significant (*P* = 0.032, Fig. 6T); there was no significant difference in the bilateral scores of the same age group (*P* > 0.05).

### TFJ Pressure Measurement

**Fig. 7:**
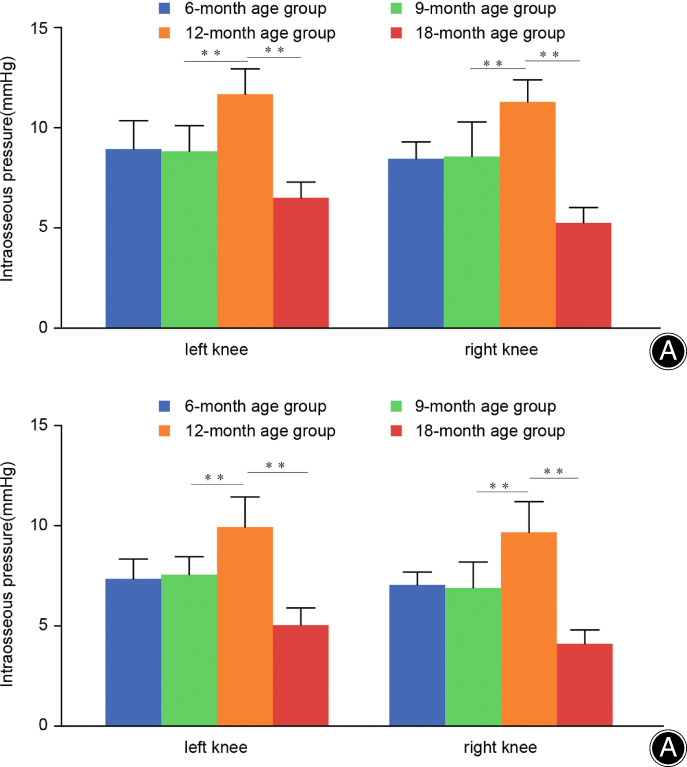
IOP of distal femur (A) and proximal tibia (B) in each age group. (Values shown are means ± SD. Double asterisk (**) indicate significant differences (*P* < 0.05) at right knee or left knee, respectively)

With the increase of age, the IOP of DF and PT increased first and then decreased, which reached its highest in the 12‐month age group (DF IOP = 11.536 ± 2.507 mmHg, PT IOP = 9.861 ± 3.199 mmHg), the difference was statistically significant (DF: *F* = 8.261, *P* = 0.000; PT: *F* = 8.469, *P* = 0.000). Compared with the 6‐month age group, the IOP increased slightly in the 9‐month age group, but no statistical difference (DF: *P* = 0.997; PT: *p* = 0.973); Compared with the 9‐month age group, the IOP was significantly higher in the 12‐month age group (DF: *P* = 0.019; PT: *P* = 0.019); However, the IOP in the 18‐month age group was significantly lower than that in the 12‐month age group (DF: *P* = 0.000; PT: *P* = 0.000) (Fig. 7). In the same age group, the IOP of DF was slightly higher than that of PT, and the difference was not statistically significant (*P* > 0.05).

## Discussion

### Relationship between Age and the Degree of KOA Degeneration

Dunkin–Hartley guinea pigs can be used as degenerative KOA animal model, which has been confirmed by many studies.^5^, ^6^, ^15^, ^16^ However, there are still no systematic studies on the radiological and histological changes at each stage of degeneration.

In the initial stage of this study, a wide range of ages were included from the smallest of 3 months to the largest of 18 months, in order to establish a more accurate link between different ages and degeneration. In this study, We first observed in Dunkin–Hartley guinea pigs that weight gain was concomitant with age, which perfectly matched the epidemiology of degenerative KOA.^15^, ^16^

For the X‐ray staging of KOA, Kellgren–Lawrence staging is usually used, and the main observation indicators include joint space, osteophytes, subchondral bone sclerosis, and whether the bone ends are deformed.^11^ Although the current X‐ray changes of KOA in Dunkin–Hartley guinea pigs are limited to morphological descriptions, because the X‐ray changes of tibiofemoral joint OA in Dunkin–Hartley guinea pigs are in good agreement with the Kellgren–Lawrence staging, we adopted this staging to evaluate. The significant differences between the 3‐month and 6‐month age groups were not observed in the K‐L classification, subchondral bone structure, intra‐articular and intramedullary signal intensity analysis. Since the guinea pig bone matured after 6 months, the 3‐month age group was excluded in the following experiment in order to eliminate the potential effect of bone growth on the changes of articular cartilage and subchondral bone.

According to radiographic and histological findings, the double TFJ of Dunkin–Hartley guinea pigs exhibited progressive degenerative aggravation from the6‐month to the18‐month age group, which with good bilateral consistency. So, it is reasonable to use this range as the classification basis for studying different degeneration stages of KOA in Dunkin–Hartley guinea pigs, and it is reliable to design the bilateral TFJ as self‐controlled experiments.

The knee joint was normal or mild degeneration in 6‐month age group, which was due to K‐L classification, and MRI showed non‐specific synovitis, although Micro‐CT can detect tiny osteophytes, there was no typical subchondral bone cyst formation, and the imaging findings were consistent with histological findings and Mankin's scores. In the 9‐month age group, the knee was slightly degenerated due to the K‐L classification, and in addition to the synovitis, MRI showed non‐specific high‐signal at the DF and PT, the signals were more diffuse. This performance echoed the density‐reduced area of the corresponding part of micro‐CT, and the articular cartilage showed mild degeneration. In the 12‐month and 18‐month age groups, micro‐CT showed a subchondral bone cystic area, which was more common in the medial condyle. The typical one was seen in the 18‐month age group. There was no bone structure remaining inside, but subchondral bone collapse fusion. MRI showed the small circular signal of the corresponding area had changed from low to high. Histological changes from the 12‐month to 18‐month age groups reflected an alternative process from reduction of surface and intermediate layer of chondrocytes, matrix decreased to deep chondrocyte proliferation. Based on the above evidence, the knee joint was moderately degenerated in the 12‐month age group, and was attributed to severe degeneration in the 18‐month age group.

The pathological changes of the tibiofemoral articular cartilage and subchondral bone of Dunkin–Hartley guinea pigs at different ages completely covered the pathological process of degenerative KOA. Selecting the appropriate guinea pig age range to study the different stages of KOA is not only conducive to the standardization of the age, but also to reduce the systemic bias caused by the different age choices.^5^, ^7^

### Subchondral High Signal Area and Subchondral Bone Cyst

Subchondral bone includes subchondral cortical bone plate and cancellous bone under the bone plate. However, the subchondral bone plate is adjacent to the calcified layer of articular cartilage, and its biomechanical properties are weaker than the cortical bone of diaphysis. Below the subchondral bone plate is a porous, metabolically active cancellous bone. As KOA progresses, the calcified cartilage layer continues to thicken, the volume of articular cartilage decreases, and the subchondral bone proliferates and hardens.^17^, ^18^, ^19^, ^20^

The high signal zone in the subchondral bone, BMLs, refers to the edema signal located in the subchondral cancellous bone of T2 liposome phase. When the range of BMLs in the subchondral bone is limited, it is spatially identical to the subchondral bone cyst. The emergence of BMLs reflects abnormalities in subchondral bone metabolism. Histological studies confirmed that BMLs were necrotic intramedullary cells and hyperplastic fibrous connective tissue.^21^ Regional subchondral trabecular bone necrosis, collapse and hyperplasia corresponded to the degeneration of the upper articular cartilage spatially.^18^, ^22^, ^23^, ^24^, ^25^ The intensity and extent of abnormal signal area in subchondral bone was positively correlated with the degree of articular cartilage degeneration.

Our study found that with the increase of age, BMLs in subchondral bone marrow and subchondral bone cysts appeared in the corresponding sites of MRI and micro‐CT, and the time consistency appeared in the 12‐month and 18‐month age groups. At that time, the articular cartilage degeneration was at the moderate and severe stage. In the 9‐month age group, the MRI subchondral bone abnormality signal was diffuse but atypical. Micro‐CT scan showed no regular shape of sclerotic bone cyst in the subchondral bone, and there was only a subchondral trabecular collapse fusion.

The collapse of the subchondral trabecular bone, the formation of bone cysts, the appearance of BMLs in the subchondral bone all corresponded to the degree of articular cartilage degeneration. It was fully consistent with the pathological changes of subchondral bone of degenerative KOA of humans. This study, on the one hand, provides powerful laboratory evidence for the use of Dunkin–Hartley guinea pigs as a degenerative KOA animal model. On the other hand, it indirectly suggests that the subchondral bone plays a crucial role in the pathological process of articular cartilage degeneration.

### Characteristics of IOP Changes in Degenerative KOA Model

IOP is an important physiological indicator describing the state of microcirculation within the bone. Bone tissue is a relatively closed system, and the maintenance of IOP depends on the balance between arterial perfusion and venous return. The volume change of the medullary cavity itself, like a reservoir, plays a role in regulating the basic capacity.

Our results showed that there was no significant difference in IOP between the DF and the PT of Dunkin–Hartley guinea pigs; the IOP of the DF was slightly higher than the PT, and the difference was not statistically significant. Although there is no statistical difference, there may be physiological significance. This difference may be related to the relatively abundant circulating blood volume in the trabecular sinus of DF. In addition, this difference may also have pathological significance. When the IOP of DF is higher than that of PT, it may cause hydrodynamic obstruction to the blood flow in the proximal trabecular bone of the tibia, the blood circulation disorder in the trabecular bone of PT, thereby a series of pathophysiological changes in the PT are further produced.

With the increase of age, the IOP of the DF and PT showed an upward trend first, the IOP in the 9‐month age group was higher than that in the 6‐month age group, but it was not statistically significant. It peaked at 12 months of age and fell rapidly at 18‐months of age. The change of IOP first increased and then decreased, which was in a good agreement with the subchondral bone changes indicated by Micro‐CT of each corresponding age group. The thickness and density of subchondral bone and cancellous trabeculae increased with age, and the direct result was that the mechanical elasticity of bone material and intramedullary space were both reduced. If the perfusion volume and the outflow amount are constant, the IOP will inevitably increase gradually. When the space decreases beyond the buffer range of the perfusion amount, the magnitude of increase of the IOP will increase significantly; If the subchondral bone proliferates and collapses, the volume of the medullary cavity will be reduced to a certain extent, which restrain the intra‐osseous blood perfusion and further reduces the IOP. This is also the reason for the sharp decline in 18‐month‐old IOP in this study.

The IOP measurement site was chose at the attachment point of the medial collateral ligament to femur and tibia for the following reasons: (i) the soft tissue inside the knee is relatively weak, and the anatomy is easy to be revealed; (ii) there is no important neurovascular structure in the surgical approach; (iii) there is basically no bleeding during the operation, which can avoid the inaccurate measurement of IOP caused by blood loss; and (iv) the anatomical position of the medial collateral ligament of the knee is constant, which can avoid the measurement error of IOP due to non‐standardization of the puncture point, and the repeatability is good.

### Histological Characteristics of TFJ Articular Cartilage

Our study found that with the growth of Dunkin–Hartley guinea pigs, chondrocytes of surface and intermediate layer of TFJ cartilage gradually decreased, however, the chondrocyte of deep layer proliferated. The acidic cartilage matrix component was progressively degraded, and the type 2 collagen fiber was reduced in the expression due to chondrocyte proliferation, and the layer disappeared. The calcified layer of cartilage was continuously thickened, and the tidal line position moved up. All of the above manifestations are the same as the classic articular cartilage degeneration pattern.

Articular cartilage fissures could be limited to the superficial and middle layer, or could be located in the deep layer alone, even in the subchondral bone plate and calcified layer cartilage, and the sinusoids invaded the deep region of the articular cartilage. This phenomenon reflected the changes in physical and chemical properties and biomechanical properties after chondrocytes and matrix components of the degenerative articular changed. Under the combined action of shear stress and axial pressure, the deformation ability decreased and laceration occurred in different parts.

MMP13 belongs to the family of matrix metalloproteinases (MMP), which are closely related to inflammatory responses. MMP13, also known as collagenase‐3, is mainly produced by chondrocytes, synovial cells, osteoblasts and neutrophils. The ability of MMP13 is to degrade type 2 collagen in articular cartilage which is much higher than other collagenases in MMPs. More importantly, MMP13 is capable of self‐catalytic activation and is central to other members of the activated MMPs family. Therefore, the small changes in MMP13 level or increased activity can amplify the decomposition of downstream protease of other MMPs.^26^, ^27^

Comparing the high expression sites of MMP13 and the degradation sites of type 2 collagen in tibiofemoral articular cartilage of Dunkin–Hartley guinea pigs, which were found in good spatial consistency. In the 18‐month age group, MMP13 was not only expressed in the whole layer of articular cartilage, but more particularly, MMP13 appeared to deposit along the tidal line. the tidal line is the site of the non‐calcified cartilage layer anchored to the calcified cartilage layer. Histologically, it is a marker separating the calcified cartilage layer and the non‐calcified cartilage layer. This deposition phenomenon is very likely to clarify and predict the pathological mechanism of full layer cartilaginous exfoliation.

### Strengths and Limitations

The Dunkin–Hartley guinea pigs of different months (6, 9, 12, 18) were used as the primary degenerative KOA animal model. It was confirmed by radiology and histology that the related degeneration scores showed a step‐by‐step progression in each month‐age group, can completely cover the natural pathological process of KOA; within each group, the difference in pathological changes of KOA is small. Therefore, relatively reliable and well‐contrasted experimental data can be obtained when this month‐age series is used in the study of KOA in different degeneration stages.

In Dunkin–Hartley guinea pigs at different ages, the intraosseous pressure of the distal femur and proximal tibia showed a trend of increasing and then decreasing with the aggravation of KOA degeneration. Combined with the structural changes of the gradually increasing density of the subchondral bone of KOA The presence of edema‐like BMLs in the subchondral bone can satisfactorily explain the changes in intraosseous pressure. The positive correlation between articular cartilage and the pathological degeneration of subchondral bone reveals that intraosseous pressure plays a key role in during the pathogenesis of articular cartilage and subchondral bone.

Since the tibiofemoral joint of Dunkin–Hartley guinea pigs is very small, the lesion area displayed by MRI is smaller, so it is difficult to accurately measure the size of the lesion. Therefore, we only used qualitative analysis of the lesion area. However, the follow‐up histological examination in this study has made up for this indirectly. At present, there is no relevant report about MRI score on knee joints of small animals such as guinea pigs. Therefore, a simple scoring system was developed for MRI quantitative assessment of KOA in our study, which was relatively simple, intuitive and high operability, but needed further verification and improvement. Also, only the morphological description of micro‐CT imaging changes was described in the ROI. Due to the large differences in organizational morphology of interest regions, not a single suitable 3D modeling method was found to accurately calculate data such as BV/TV.

In this study, the quantification such as Type 2 collagen and BMP13 were not analyzed by image software in immunohistochemical staining, which might show little significance.

Changes in patellofemoral KOA were not included in this study. There are two reasons: (i) The patella of the DH guinea pig is very small, so it is difficult to measure the intraosseous pressure; and (ii) among the pathological changes of KOA, the change of tibiofemoral joint is the most important pathological change.

### Conclusion

Dunkin–Hartley guinea pigs of 6 to 18 months old could be used as reliable KOA animal model which completely covered the different pathological stages of natural degeneration of KOA through imaging and histological studies for the first time. The progress of KOA was mild at 6‐month age, moderate at 9–12 months, and severe at 18‐month age. The self‐controlled study confirmed that the difference in bilateral KOA progress was small in Dunkin–Hartley guinea pigs. The characteristics of IOP changes in tibiofemoral joint were in good agreement with the pathological progress of KOA, revealing the correlation between IOP and pathological changes of articular cartilage and subchondral bone.

## Authors' Contributions

Experiment design and supervision were performed by Shuo Wang, Jianxiong Ma, Yuan Xue and Xinlong Ma. Animal experiment, data collection and analysis were performed by Shuo Wang, Wei Liu, Lei Zhang and Aixian Tian. The first draft of the manuscript was written by Shuo Wang, Xingwen Zhao and Hongchao Huang. Final edition and subsequent revisions was completed by Shuo Wang and Xingwen Zhao. All authors commented on previous versions of the manuscript. All authors read and approved the final manuscript.

## Ethics Approval

The animal study was reviewed and approved by the Experimental Animal Care and Use Committee (EACUC) of Tianjin Hospital. Written informed consent was obtained from the owners for the participation of their animals in this study.

## Supporting information


**Figure S1.** The anatomical region of interest (ROI). The coronal plane was marked from the anterior calcified cartilage of the meniscus to the sesamoid of posterior‐superior femoral condyle. The sagittal plane was marked between the medial and lateral crest of femoral patella surface.

**Table S1.** Kellgren–Lawrence classification
**Table S2**. MRI simple scoring system for KOA of Guinea pig
**Table S3**. Mankins score system.
