# Supplementary material for: The Osteoarthritis Natural Progress and Changes in Intraosseous Pressure of the Guinea Pig Model in Different Degeneration Stages
Source: Orthop Surg. 2022 Sep 28;14(11):3036–46. doi: 10.1111/os.13496 (PMC9627048; doi:10.1111/os.13496)
Supplement: Supplementary file 1 — Figure S1. The anatomical region of interest (ROI). The coronal plane was marked from the anterior calcified cartilage of the meniscus to the sesamoid of posterior‐superior femoral condyle. The sagittal plane was marked between the medial and lateral crest of femoral patella surface. [file OS-14-3036-s001.doc]

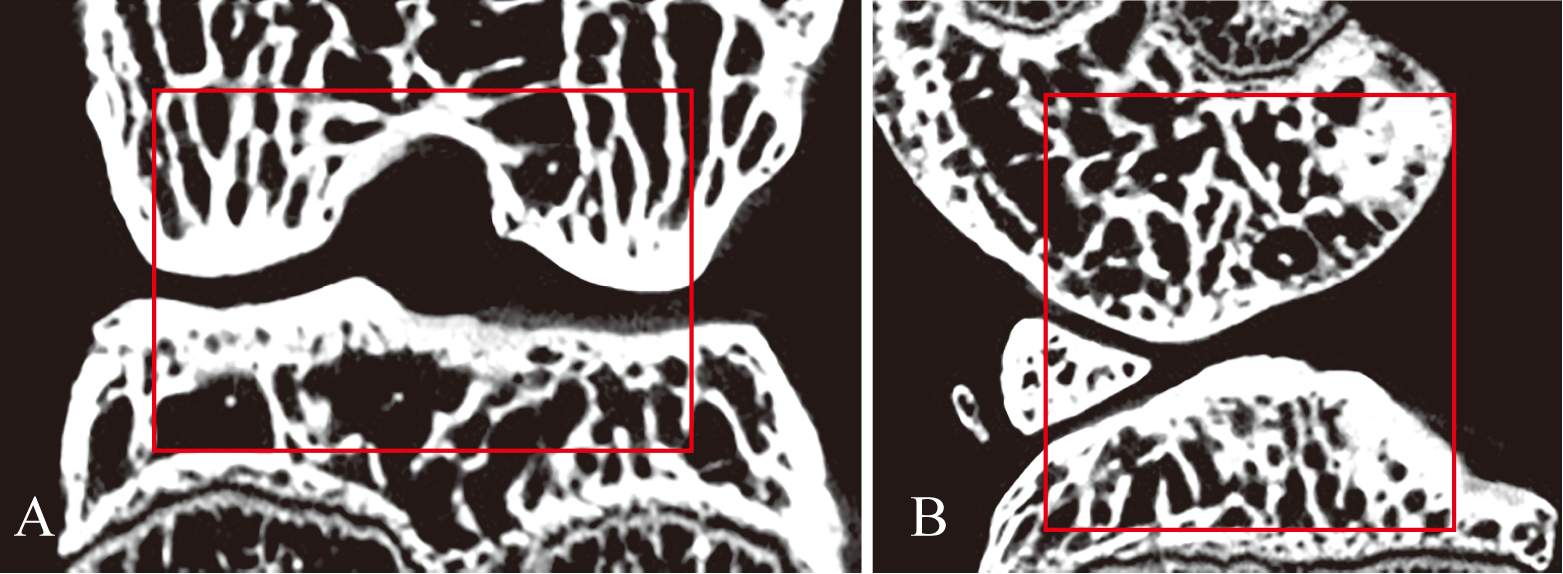


Supplementary Fig1 The anatomical region of interest (ROI). The coronal plane was marked from the anterior calcified cartilage of the meniscus to the sesamoid of posterior-superior femoral condyle. The sagittal plane was marked between the medial and lateral crest of femoral patella surface.
