# Supplementary material for: The Osteoarthritis Natural Progress and Changes in Intraosseous Pressure of the Guinea Pig Model in Different Degeneration Stages
Source: Orthop Surg. 2022 Sep 28;14(11):3036–46. doi: 10.1111/os.13496 (PMC9627048; doi:10.1111/os.13496)
Supplement: Supplementary file 2 — Table S1. Kellgren–Lawrence classification Table S2. MRI simple scoring system for KOA of Guinea pig Table S3. Mankins score system. [file OS-14-3036-s002.doc]

Supplementary Table 1 Kellgren-Lawrence classification

| Grade | Radiology description |
| --- | --- |
| 0 | No radiographic features of osteoarthritis are present |
| 1 | A doubtful narrowing of joint space and possible osteophytes |
| 2 | Presence of osteophytes and possible joint space narrowing on the anteroposterior weight-bearing radiograph |
| 3 | Multiple osteophytes, definite joint space narrowing, sclerosis and possible bony deformity |
| 4 | Large osteophytes, marked joint space narrowing, severe sclerosis, and definitely bony deformity |

| **Supplementary Table 2 MRI simple scoring system for KOA of Guinea pig** | | |
| --- | --- | --- |
| Pathological manifestations | Signal characteristics | Score |
| Synovitis | None | 0 |
|  | High signal presence | 1 |
|  | Significantly high signal | 2 |
| Bone marrow edema and/or cystic changes | None | 0 |
|  | High signal presence | 1 |
|  | Significantly high signal | 2 |

**Supplementary Table 3 Mankins score system**

| Parameter | Grade | Description |
| --- | --- | --- |
| Articular Cartilage Structure | 0 | Normal, smooth, uninterrupted surface |
|  | 1 | Mild surface irregularities |
|  | 2 | fissures and/or loss of cartilage in the superficial zone |
|  | 3 | fissures and/or loss of cartilage extending into the middle zone |
|  | 4 | fissures extending into the deep zone and/or loss of cartilage to deep zon |
|  | 5 | Fissures or loss of cartilage extending to the zone of calcified cartilage |
|  | 6 | Full-thickness cartilage loss |
| Proteoglycan Content (staining by toluidine blue) | 0 | Uniform throughout articular cartilage |
|  | 1 | Decreased in superficial zone only |
|  | 2 | Decreased in superficial and middle zones |
|  | 3 | Decreased in all 3 zones |
|  | 4 | No staining |
| Cellularity | 0 | Normal (1/2 cells) |
|  | 1 | Diffuse/slight hypercellularity |
|  | 2 | Regions of hypercellularity and clustering |
|  | 3 | Diffuse hypocellularity |
| Tidemark Integrity | 0 | Intact/single tidemark |
|  | 1 | Crossed by vessels/duplication of tidemark |
